# Challenges of Developing Novel Vaccines With Particular Global Health Importance

**DOI:** 10.3389/fimmu.2020.517290

**Published:** 2020-10-14

**Authors:** Penny M. Heaton

**Affiliations:** Bill & Melinda Gates Medical Research Institute, Cambridge, MA, United States

**Keywords:** vaccine development, low resource areas, tuberculosis, malaria, HIV/AIDS, infectious diseases, respiratory syncytial virus, shigella

## Abstract

Six of the top ten leading causes of death in low resource settings could potentially be prevented by vaccination. Development of vaccines for individuals in these populations is difficult because of the biological complexity of the prevalent pathogens and the challenges inherent to development of any vaccine. This review discusses those challenges and promising advances to address them and highlights recent progress in development of vaccines against several pathogens of interest.

## Introduction

Vaccines are among the greatest health interventions known to humankind, second only to safe water with regard to number of lives saved. Although taken up widely in high-income countries (HIC) from the time the first vaccine was produced against smallpox, vaccination in low- and middle-income countries (LMIC) where mortality from infectious diseases is highest was not widespread until the World Health Organization implemented the Expanded Programme on Immunization (EPI) in 1974. Building on the success of the smallpox eradication efforts, the EPI recommended vaccines to protect against six diseases including tuberculosis, diphtheria, tetanus, pertussis, measles, and poliomyelitis. Five additional vaccines have since been recommended to prevent hepatitis B, *Hemophilus influenzae* type b, pneumococcus, rotavirus, and rubella. The impact has been staggering including near eradication of poliovirus and dramatic reduction in childhood mortality with deaths cut by more than half. Despite the amazing progress, more breakthroughs are needed; approximately three million individuals still die of vaccine-preventable diseases each year and infectious diseases such as lower respiratory infections, tuberculosis (TB), HIV/AIDs, diarrheal diseases, malaria, and those associated with preterm birth make up six of the top ten leading causes of death in low-income countries (1). The purpose of this review is to highlight the challenges of vaccine development against these diseases and to share new advances that give hope it won't take another half century to scrub infectious diseases from global mortality statistics.

## General Challenges of Vaccine Development

**Figure 1:**
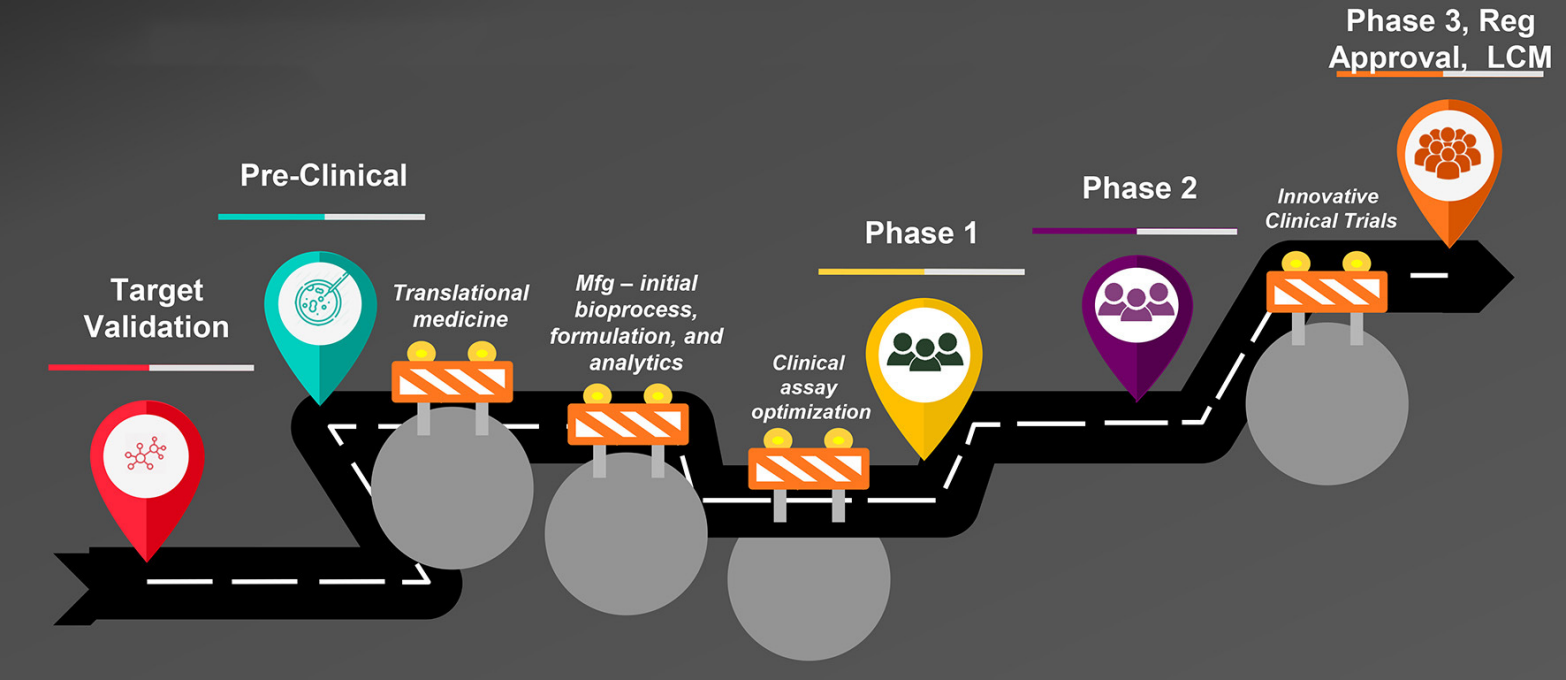
Steps in vaccine development. Mfg, manufacturing; Reg, regulatory; LCM, life cycle management.

Vaccine development is uniquely challenging when compared with development of other product modalities such as small molecules. Figure 1 is an illustration of the steps in vaccine development from creation of the vaccine candidate through preclinical and clinical studies to regulatory approval and lifecycle management. Typical bottlenecks that delay progression through these steps are well known. First is the so-called “valley of death,” the transition from the laboratory to clinical-trial-enabling activities. The most notorious hurdles responsible for vaccine development failures or delays during this transition are two-fold: the complexity of development of the manufacturing process, formulation, and analytical assays and the difficulty of clinical assay optimization. A vaccine construct is not a laboratory-synthesized chemical moiety but a modified live virus or bacterium–or a component thereof–that is intended to induce a protective immune response in a healthy individual. Therefore, the manufacturing process for vaccines inherently involves growth and modification of live organisms or their components, or recombinant protein expression in a live cell line. Biological entities are highly variable, yet the process must be optimized to make vaccine consistently with pre-specified characteristics and purity and commercially viable yields. A formulation in which to suspend the modified organism or protein is needed that is appropriate for parenteral or oral administration and that stabilizes the entity to support adequate shelf life. Finally, analytical assays to characterize the vaccine and measure the “potency” or dose level that reflects the quantity of the relevant immunologically active component(s) must be developed and validated. Each of these activities is complex requiring the integrated work of experts from many disciplines and can take several years to accomplish.

The second hurdle in the valley of death is optimization of clinical laboratory assays to measure the immune response induced by the vaccine. Vaccine concentration and pharmacodynamics cannot be measured directly; the biological effect of interest in vaccine clinical trials is the ability of the vaccine to induce an immune response, typically antibody, that protects against infection and/or disease. Clinical laboratory assays are required to measure both antibody quantity and quality (i.e., is the antibody “functional,” killing the pathogen of interest?) as well as other measures of immune stimulation, such as CD8+ T cell activation. The immunologic assays are first evaluated in preclinical studies designed to show a correlation between vaccine dose level and immune response. Ideally, these preclinical studies would also show efficacy in challenge studies and support selection of a range of doses to be evaluated in clinical studies. Immunologic assay results are often highly variable, particularly those measuring functional responses, but they must be optimized to provide consistent results with appropriate positive and negative controls in order to receive regulatory approval for use in clinical trials.

As candidate vaccines progress to clinical trials, another universal challenge is that the target populations are healthy individuals. Therefore, the benefit-risk assessment differs from that of a therapeutic agent that is being administered to an ill patient; the bar for demonstrating safety is high and the safety profile must be acceptable to regulators, ethics committees, policymakers, parents, and individuals receiving the vaccines. Large clinical trials with several thousand subjects are needed pre-licensure to establish the safety profile and inform the benefit-risk assessment for using the vaccine broadly. Further, safety must continue to be monitored post-licensure for detection of uncommon adverse events that may not have been detected in pre-licensure studies.

## Challenges of Vaccine Development in Low Resource Settings

### Technical Challenges

The challenges of vaccine development for low resource settings start with this rather daunting set of baseline challenges overlaid with technical challenges stemming from the significant biological complexity of the target pathogens of interest, particularly those causing TB, malaria, and HIV/AIDs. With rare exceptions, vaccines constructed from whole or modified organisms or surface proteins have had low or no efficacy against these diseases in clinical trials. As summarized in Table 1 and discussed in the sections that follow, the reasons are multifactorial. First, antigen selection has been convoluted by the complex lifecycles of *Mycobacterium tuberculosis* (Mtb), *Plasmodium* species, and Human Immunodeficiency Virus (HIV) with each having the ability to establish chronic, asymptomatic infection and presumably escaping immune detection for at least part of the cycle. Antigen selection is further complicated by genetic and antigenic variability across strains including geographic variability and *in vivo* evolution of strains after infection has been established, underscoring the need to search for conserved antigens or bioengineer mosaics that will cover the majority of strains. Although functional antibody is typically the mechanism by which vaccines induce protection, other immune responses may be important for these pathogens such as binding antibody and classical and non-classical T cell responses, and the desired response may differ by lifecycle stage. Further, for those vaccines which have shown at least limited efficacy in clinical field trials, protection is short-lived without sufficient effector memory. Improved antigen “quality” and/or novel adjuvants are needed to extend the duration of efficacy and clinical benefit.

**Table 1:** Vaccine development challenges: overall and unique to low resource settings.

**Vaccine development challenges**	
**All vaccines**	**Unique to TB, malaria, and HIV/AIDs vaccines**
• Manufacturing–bioprocess, formulation, and analytical development	• Complex life cycle of target pathogens–antigen(s) selection difficult
• Optimization of clinical immunologic assays	• Protective immune response unclear
• Large clinical trials required to evaluate safety in healthy individuals	• Poor memory responses with rapidly waning efficacy
	• Regulatory, Ethical Committee, and clinical trial infrastructure limitations for large studies involving novel technologies
	• Insufficient financial resources for development
	• No/limited high-income country markets

### Clinical Trial Implementation Challenges

After the initial technical development challenges are addressed and favorable phase 1 safety and immunogenicity data generated, vaccine development typically progresses to a phase 2 “proof-of-concept” (PoC) study in the target population to further evaluate safety and to generate the initial evidence that the vaccine protects against the disease of interest. For TB, malaria, and HIV vaccines, the desired protective immune response is unclear. Therefore, large phase 2 studies designed to show a statistically significant signal of efficacy i.e., reduction in disease in vaccines as compared with controls are required to demonstrate PoC. Dependent on the disease burden, sample sizes of several hundred to thousands may be needed to accrue enough disease cases to make this comparison with statistical rigor. The silver lining is that these large PoC studies also present the opportunity to identify the vaccine-induced immune response(s) that correlate with disease prevention, which can significantly simply further development.

As will be discussed in the sections that follow, strategies have been employed for these high-risk vaccine programs to reduce the probability of late stage development failures. For TB vaccines, PoC may be shown through smaller “prevention of infection” (POI) studies conducted utilizing a biomarker. Initial efficacy data for malaria vaccines may be shown in challenge studies using the Controlled Human Infection Model (CHIM). For HIV, immune responses similar to those previously identified to correlate with protection may gate progression to larger efficacy studies. Although each of these approaches provides supportive data for decisions on further development, ultimately large studies showing a vaccine prevents disease as compared with a control are required.

Even in the best of circumstances, large studies with these safety, efficacy, and immunogenicity objectives are difficult to implement. Clinical trial sites with robust infrastructure, sophisticated laboratories for processing clinical samples, and highly trained personnel are needed. Further, the national regulatory authorities and ethics committee members who approve the studies to move forward must understand the approach to vaccine design and manufacturing, the implications of preclinical study results and immunologic assay performance, and the PoC study design rationale and intended outcomes to appropriately assess benefit versus risk for their populations. Finally, early engagement with local community leaders is essential to address potential concerns about study conduct and avoid myths and rumors that may circulate once the study has started. Each of these challenges is amplified even further for pivotal, confirmatory phase 3 trials, which are larger still (tens of thousands).

Although initially conduct of large PoC and pivotal phase 3 studies in low resource settings may seem an impossible task, they are feasible with the right partners and planning. Stimulated by the emergence of HIV and further supported by global public health malaria and TB research initiatives, several academic, government, and non-government organizations have built clinical trial site and laboratory capacity in low resource settings and additional efforts to close research capacity gaps are ongoing. Further, approaches have been defined for following and retaining participants considered high risk because of co-morbidities not encountered in high resource settings or complex social circumstances. The WHO provides guidance, training, and support for national regulatory authorities in its Member States to review and approve clinical trial applications (2). Because of increasing medical product research in Africa, the WHO established the African Vaccine Regulatory Forum (AVAREF) in 2006, a platform that brings together national regulatory authorities and ethics committees on the African continent to improve and harmonize review practices and ensure timely decisions on vaccine clinical trial applications. Several vaccines including meningococcal, rotavirus, pneumococcal, and ebolavirus vaccines have been approved utilizing this platform (3).

### Funding, Introduction and Commercial Challenges

Creation of a sustainable model for development, introduction and commercialization of products intended almost solely for individuals in low resource settings is also an ongoing unmet medical need. Vaccine development is expensive with estimated costs from research and discovery to registration between 200 and 500 million US dollars (4). This estimate accounts for vaccines that are abandoned during development. In the case of vaccines for TB, malaria, and HIV, it is likely the development costs for a single vaccine (not including failures) will rise above the 500-million-dollar ceiling because of the technical complexity and large clinical trials required as referenced above. With little to no market in high resource settings either because of low disease prevalence (TB and malaria) or the availability of other viable alternatives (HIV), there is little incentive for pharmaceutical and biotechnology companies to invest in development of these vaccines. Government and philanthropic organizations are supplementing private sector funding and are the sole funders for development in many cases but cannot bear the funding burden alone. Development funding from all stakeholders is needed.

Vaccine development costs may be further amplified by post-approval requirements for additional studies before broad recommendations are made. Large safety and effectiveness studies are increasingly being required for vaccines with modest efficacy to more fully assess the benefit-risk profile. For vaccines requiring multiple doses or given outside of existing schedules, an evaluation of how well the vaccine integrates into existing immunization programs is a good practice. Although critical, these activities may be as costly as full development programs. To reduce timelines for broad vaccine introduction and manage ever-increasing vaccine development costs, a system for the World Health Organization and associated vaccine advisory groups to provide early feedback on the data necessary to support recommendations is needed so that these considerations can be incorporated into pre-approval phase 3 studies when feasible.

Assuming funding is available and these vaccines progress favorably through phase 3 studies and regulatory approval, commercialization partners will be needed for manufacturing and widespread distribution. It is possible that commercialization partners may include large pharmaceutical companies but are more likely to be vaccine companies emerging from low- and middle-income countries with a business model that embraces vaccines sold at high volumes but with a low profit margin. The manufactured vaccine must then be made available to those who need it most, individuals in high prevalence countries. Again, an innovative business model will be needed to support countries, such as the one utilized by Gavi, the Vaccine Alliance, in which countries contribute a proportion of the vaccine costs and receive supplemental support from Gavi until they can fully support the vaccine costs on their own (5).

The sections that follow summarize the status of development of vaccines against TB, malaria, HIV, and other diseases. Pathogen-specific technical challenges and recent technological advances and clinical trial results that provide optimism for the future are discussed.

## TB Vaccines

*Mycobacterium tuberculosis* (Mtb) has evolved with humankind over the last 40,000 years, harnessing the immune system to its advantage. An estimated one quarter of the world's population is infected with Mtb. Although only 10% of those infected develop symptomatic disease, because of the high Mtb prevalence, this translates to an estimated 10 million cases and 1.4 million deaths annually (6). Tuberculosis is a disease of poverty; deaths peaked in the United States (US) and Europe in the early years of the industrial revolution and all but disappeared with improved living and working conditions. Currently, most TB-associated deaths occur in low- and middle-income countries with two thirds of the disease burden occurring in eight countries including India, China, Indonesia, the Philippines, Pakistan, Nigeria, Bangladesh, and South Africa (6). Unlike in high-income countries where TB occurs primarily in the elderly, in low resource settings the majority of lives taken are young and middle-aged adults, the most productive memories of society (7). There is currently no TB vaccine for these individuals, who are also the primary transmitters of Mtb.

The technical challenges for TB vaccine development are not to be underestimated. The unique ability of this organism to live amicably with its host for several years to a lifetime raises basic questions about which Mtb components should be targeted as antigens to be included in a vaccine. Further, Mtb appears to express different antigens at different stages of the disease cycle i.e., infection, subclinical disease, and clinical disease. However, the immunologic mechanism for killing the organism across different stages of disease is unclear. Given it is plausible that reduced risk of disease relies on controlling Mtb growth and prevention of sustained infection, investigations into immunologic correlates of risk and protection should cast a wide net. Natural cohort studies and studies of failed vaccines support different potential mediators of immunity including Mtb-specific T cells, functional antibody, and innate immunity via non-classical T cell responses (8–10).

Questions regarding the relationship between prevention of infection (POI) and prevention of disease (POD) shape the current global TB vaccine development strategy as scientists push to simultaneously understand Mtb immunopathogenesis and develop safe and efficacious vaccines. While a dual approach is appropriate given the magnitude of TB morbidity and mortality globally, in practice it means conducting large and complex clinical trials. Although a validated biomarker, Mtb-specific gamma interferon release, simplifies studies evaluating vaccines for POI, currently the only means to show a vaccine prevents disease is through evaluation of clinical TB disease cases in controlled clinical trials, which require many thousands of subjects from low resource areas where the prevalence of TB is high.

Despite the technical and clinical trial complexities, the biggest challenge for TB vaccine development may be non-technical. Because TB was nearly eliminated by the middle of the twentieth century in high- and middle-income countries through improved sanitation and drug therapy, resources for development of better TB vaccines have been significantly limited, with some estimates suggest a funding shortage of ~150 million US dollars annually (11). Although the HIV epidemic and the emergence of multi-drug resistant TB has brought renewed interest to the field, key epidemiological and immunological hypotheses are only now being elucidated. If the dual approach of studying immunopathogenesis at the preclinical and early clinical level while simultaneously pursuing clinical studies to evaluate whether available vaccine candidates can prevent Mtb infection and/or disease is to be realized, considerably more funding is needed for the global TB vaccine envelope.

### Key Highlights From the Current Global TB Vaccine Portfolio

**Figure 2:**
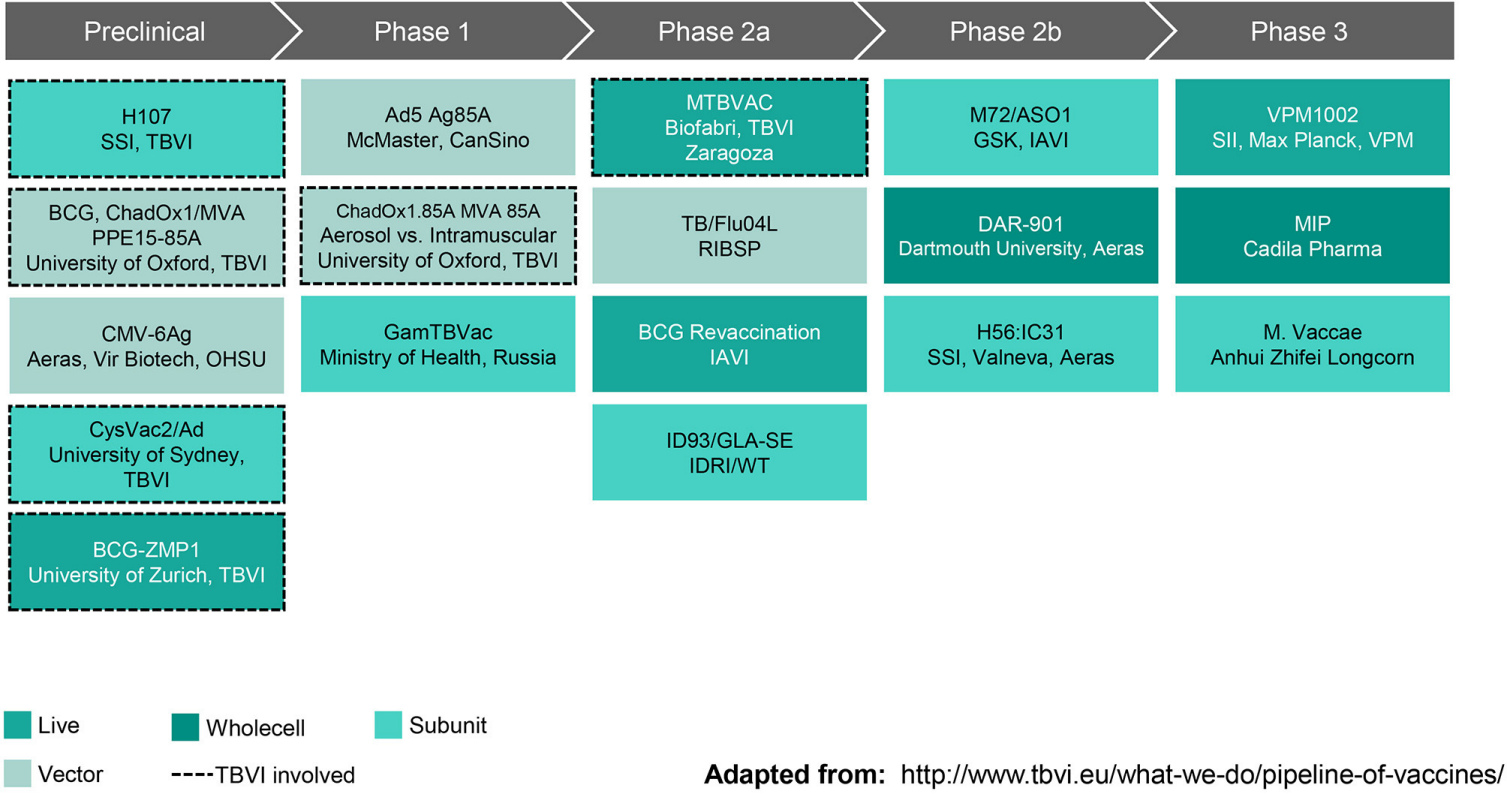
Global Pipeline, TB Vaccines for Adolescents & Adults (October 2019). SSI, Statens Serum Institut; TBVI, TuBerculosis Vaccine Initiative; ChadOx1/MVA, Chimpanzee Adenovirus, Oxford University #1 and Modified Vaccinia virus Ankara; PPE, family of Mtb genes; CMV-6Ag, cytomegalovirus vector expressing six Mtb antigens; OHSU, Oregon Health & Sciences University; CysVac2/Ad, Mtb fusion protein with novel adjuvant; ZMP, zinc metalloprotease deletion mutant; Ad5 Ag85A, human type 5 adenovirus vector expressing antigen 85A; GamTBVac, two Mtb fusion proteins with a novel adjuvant; MTBVAC, rationally attenuated Mtb clinical isolate; TB/Flu04L, live recombinant influenza vectored TB vaccine; BCG, Bacille Calmette-Guerin; IAVI, International AIDS Vaccine Initiative; ID93/GLA-SE, recombinant fusion protein in a lucopyranosyl lipid A stable emulsion; IDRI, Infectious Disease Research Institute; WT, Wellcome Trust; M72/AS01, recombinant fusion protein combined with the AS01 adjuvant system; GSK, GlaxoSmithKline; DAR-901, inactivated *M. obuense*; H56:IC31, recombinant fusion protein in a novel adjuvant; VPM1002, recombinant BCG; SII, Serum Institute of India; VPM, Vakzine Projekt Management GmbH; MIP, *M. indicus pranii*. Reprinted with permission from TuBerculosis Vaccine Initiative (12).

New interventions will be needed to achieve the World Health Organization's End TB goal of reducing deaths by 95% by 2035 (as compared with 2015). A vaccine that prevents disease in adolescents and young adults, the primary transmitters driving the TB epidemic in low income countries, is essential to reaching this goal. As summarized in Figure 2, the current TB vaccine portfolio is based on a variety of different approaches including live, attenuated whole cell vaccines, replication-competent and incompetent vector-based vaccines, and subunit vaccines with adjuvants. The sections that follow highlight some vaccines that have shown the most promising clinical and preclinical data to date.

### BCG Prevention of Infection Study

The only TB vaccine currently approved and recommended by the World Health Organization is the nearly one-hundred-year-old Bacillus Calmette Guerin vaccine (BCG). It is a live, attenuated *Mycobacterium bovis (M. bovis)* that cross-protects against Mtb. The vaccine is indicated for prevention of disseminated TB in infants and young children. Since it was first administered to infants in 1921, several studies have been conducted showing variable results with efficacies ranging from 0 to 99% depending on the endpoint of the study. A meta-analysis of these studies suggests the efficacy in newborns and young children is ~50% but wanes in the first two decades of life leaving older adolescents and young adults vulnerable to infection and disease (13). Accordingly, several studies have been conducted evaluating the efficacy of a BCG booster dose in adolescents showing highly variable results and a BCG booster is not currently recommended. Several potential confounding factors have been identified with the most likely being exposure to cross-reactive *Mycobacterium* species that leads to either blocking of BCG take or masking of BCG antibody response. In 2018, a three-arm study including a TB subunit vaccine composed of a fusion protein with a proprietary adjuvant (H4:IC31), BCG revaccination, and a placebo was conducted in adolescents vaccinated with BCG at birth in the Western Cape of South Africa to evaluate efficacy to prevent Mtb infection as measured by QuantIFERON testing. Although the primary endpoint of the study, prevention of initial infection, was not met, BCG revaccination was 45% efficacious in preventing sustained infection defined as lack of QuantiFERON conversion over a 6-month period (14). These results are being confirmed in an appropriately powered follow-up study to evaluate if a BCG booster can indeed prevent infection in those previously primed with BCG in infancy.

### DAR-901 Prevention of Infection Study

The DAR-901 vaccine is a whole-cell, heat-inactivated non-tuberculous mycobacterium (*M. obuense*, previously identified as *M. vaccae*). This vaccine is highlighted because of the results of a previous phase 3 study of a parent strain of this vaccine conducted in HIV-infected individuals with a CD4 count of > 200 who had received BCG at birth. Although the study did not meet the primary endpoint of preventing disseminated TB or a secondary endpoint of “probable” TB, the study did meet a secondary endpoint preventing 39% of “definite” TB (15). This vaccine did not have a scalable manufacturing process and has since been rederived as DAR-901, which was generally safe and well-tolerated in phase 1 studies. The efficacy of the vaccine to prevent infection as identified by an interferon gamma release assay (IGRA) is currently being evaluated in a randomized, controlled study of previously BCG-immunized adolescents in Tanzania.

### M72/AS01E Prevention of Disease Study

The results of this study are some of the most promising in the history of TB vaccine development. The M72/AS01E vaccine is composed of a fusion protein (36A and 39A) formulated with AS01E adjuvant containing QS21 and MPL in a liposomal formulation. A phase 2b randomized (1:1), placebo-controlled study was conducted in 3,575 HIV-uninfected subjects 18 to 50 years of age in Kenya, South Africa, and Zambia to evaluate the efficacy of two doses of the vaccine to prevent disease in subjects already infected with Mtb as demonstrated by a positive QuantiFERON test (16). An initial analysis of this study showed the vaccine was generally safe and well-tolerated with 13.1% of subjects in the vaccine group and 6.9% in the placebo group reporting at least one grade 3 symptom. One vaccine-related serious adverse event was reported in each group. The vaccine was 54% [90% confidence interval (CI), 13.9 to 75.4] efficacious against progression to bacteriologically confirmed active pulmonary TB disease. Final analyses showed results consistent with the initial; the same magnitude of efficacy was observed after 3 years of follow-up with no differences by gender or age (17). The parent company is evaluating partners to continue its development.

### Promising Preclinical Studies–BCG by Alternate Routes; CMV Vector-Based TB Vaccines

Because protection with intradermal (ID) BCG administration is incomplete, alternative routes of administration have been studied. A recent study in rhesus macaques compared the immunogenicity and efficacy of BCG administered by ID and intravenous (IV) injection, or as an intratracheal mucosal boost (ID + IT). The study showed that BCG administered intravenously induced protection surpassing that achieved by all other routes with 5 of 6 monkeys protected (18). Although IV BCG is not practical for low income countries because of the complexity of administration and need for immunodeficiency screening beforehand, this study is important because this approach could be a valuable tool to understand immunologic mechanisms of protection against Mtb and could serve as a benchmark for protection of new vaccines in development.

Another promising preclinical TB vaccine candidate is an attenuated, replication-competent CMV vector-based vaccine selected because of the ability to induce and maintain robust CD4+ and CD8+ T-cell memory responses. Rhesus cytomegalovirus (RhCMV) vectors coding for a variety of Mtb antigens were evaluated in two independent Mtb challenge studies in rhesus macaques (19). These challenge studies showed the vaccines were 68% efficacious against pulmonary and extra-pulmonary Mtb infection as compared with unvaccinated controls. Further, 41% (14 of 34) of RhCMV/TB-vaccinated macaques across both studies showed no TB disease by computed tomography scan or at necropsy compared with 0 of 17 unvaccinated controls. These results are exceptional among TB vaccine preclinical studies. Currently, the constructs and manufacturing process are being optimized in anticipation of phase 1 clinical studies in 2020.

In summary, although the life cycle and immunopathogenesis of Mtb remains to be clarified, data from recent preclinical and clinical studies provide hope that an approved vaccine may be on the horizon in the next decade. Simultaneously with clinical trials, it is imperative that scientists continue to understand the Mtb life cycle, identify new target antigens and clarify the immunologic mechanisms of protection against infection, subclinical and clinical disease to ensure “next generation” vaccines that are even more promising than those currently moving through the TB vaccine pipeline. Sufficient funding for this work is an unquestionable roadblock; the significant mortality associated with Mtb calls for stakeholders to come together with creative funding solutions and business models to work toward the goal of ending the TB epidemic globally.

## Malaria Vaccines

**Figure 3:**
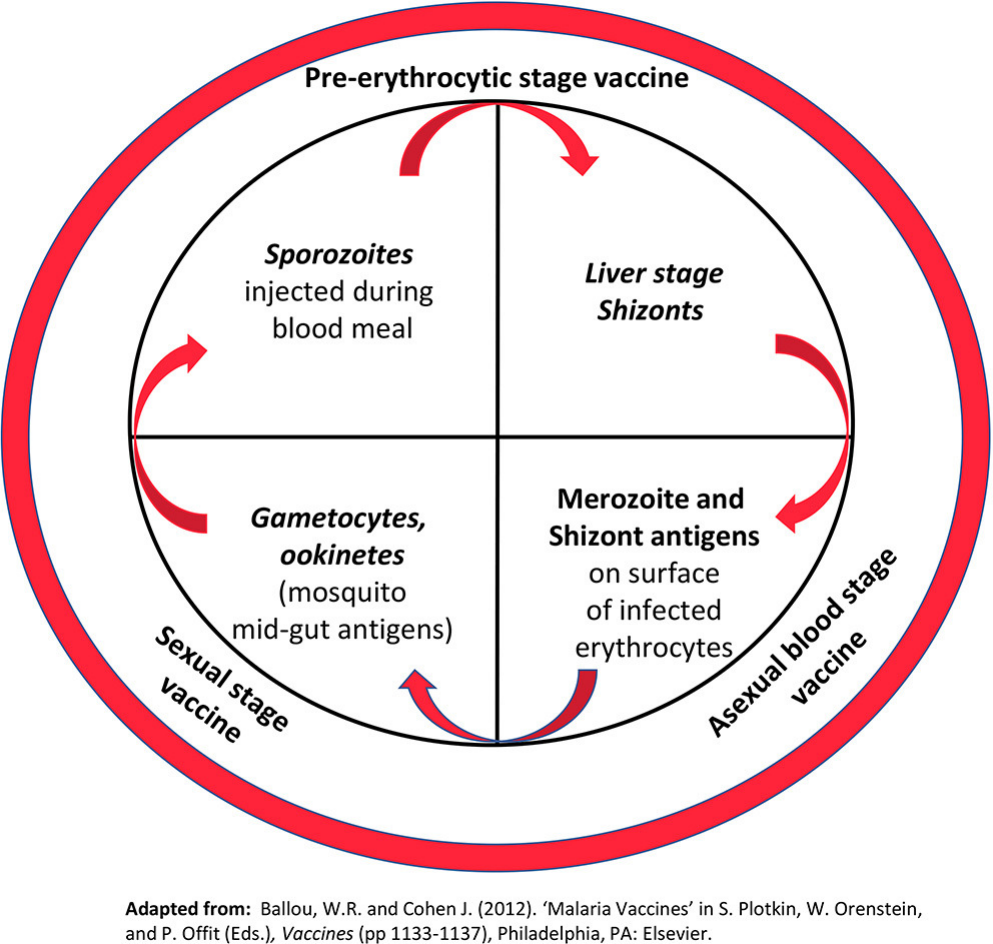
*Plasmodium* life cycle. The inner circle highlights key stages of the parasite life cycle targeted by malaria vaccines under development. Stages in italics represent biological bottlenecks where relatively few parasites (< 100) must be targeted to break the life cycle. (For comparison, the total number of merozoites/schizonts in the human host can exceed 10^12^). The outer circle shows the major classes of malaria vaccines under development Reprinted with permission from Elsevier: Ballou and Vekemans (20).

Malaria occurs from infection with *Plasmodium* parasites, transmitted to humans from the female Anopheles mosquito. The *Plasmodium* life cycle is illustrated in Figure 3. Although malaria is preventable and curable, an estimated 228 million cases occurred in 2018 leading to 405,000 deaths (21). The African Region bears the greatest burden of this disease having 93% of the 2018 cases and 94% of deaths. *Plasmodium* infections are typically asymptomatic or associated with uncomplicated disease including fever, chills, headache, nausea and vomiting, and fatigue. Severe disease occurs when infected erythrocytes sequester in blood vessels leading to major organ failure. Individuals at highest risk of infection and severe malaria are infants and young children <5 years old, pregnant women, individuals with HIV/AIDs, and non-immune migrants or travelers. Currently, prevention is best provided through vector control agents such as indoor residual spraying with insecticides and pyrethroid insecticide-treated bed nets. Antimicrobials are also available for chemoprophylaxis. In those already infected, artemisinin-based combinations are the best available therapy. These tools combined with environmental management have significantly reduced malaria morbidity and mortality. However, resistance to insecticides and antimicrobials has been a recurring problem throughout history. To ensure malaria elimination, a vaccine is needed.

No vaccines are available for widespread use. Only one vaccine has shown partial protection against malaria, RTS,S/AS01 (Mosquirix™ by GlaxoSmithKline) (22). The vaccine consists of a subunit protein—the NANP repeats and C-terminal domain of the *Plasmodium falciparum* pre-erythrocytic circumsporozoite protein (CSP) fused to the hepatitis B virus surface antigen—formulated with ASO1 adjuvant containing QS21 (*Quillaia Saponaria*, a purified saponin derivative) and monophosphoryl lipid A (MPL). The vaccine was ~50% efficacious against clinical malaria in infants 5 to 17 months of age in the initial analysis of a large phase 3 study conducted in southern Africa, but additional data showed the efficacy is low in young infants and wanes over time (23). The mechanism of protection is thought to be high antibody titers against the NANP repeats. RTS,S/AS01 was approved by European regulators in 2015 and has since been introduced in three pilot programs in Malawi, Ghana, and Kenya to evaluate safety in the context of routine use, the potential impact on reducing childhood deaths, and the feasibility of implementation in the context of a real-world setting.

The challenges of malaria vaccine development are summarized in Table 2. Like Mtb, the life cycle of *Plasmodium* species is complex and the preferred antigen(s) for vaccines are ill defined. The mechanism of immunity to *Plasmodium* species is as complex as the parasite's life cycle. Host factors are important for protection; in highly endemic areas, alterations in hemoglobin structures or certain erythrocyte-associated enzymes provide at least partial protection against infection and severe disease (24). Non-specific immune responses also limit disease progression. Natural Killer (NK) cells likely play an important role through direct action on the parasite as well as recruitment of macrophages and other cells important for early parasite control (25). Adaptive immunity including antibody and CD8+ T cells are important at different stages of the life cycle (24). Antibody appears to block the parasites traverse from one stage of the life cycle to the next including sporozoite invasion of hepatocytes, merozoite invasion of erythrocytes, and sequestration of infected erythrocytes through blocking their binding to the vascular endothelium. Antigen-specific CD8+ T cells inhibit parasite development in hepatocytes. Interestingly, the parasite also appears to take advantage of its human host by blocking development of robust antibody responses through interruption of T follicular helper cell differentiation, a potential explanation for the relative immunosuppression that has been observed in individuals with this disease (26).

**Table 2:** Malaria vaccine development challenges.

**Malaria vaccine development challenges**	
**Challenge**	**Commentary**
Immune response(s) associated with protection are not fully defined.	• Antibody to NANP repeats of the circumsporozoite protein appear to protect at high titers • Role of antibody to other proteins and cell mediated immunity is not well understood • Innate immune responses appear to impair the development of adaptive, long-term protection
Strain Variation – impact on vaccine efficacy is not well defined.	• Different *Plasmodium* species cause malaria • Strain variation occurs within species • There is antigenic variation of clones as they progress through their life cycle
Protein expression and manufacturing	• Surface proteins important for blood-stage and transmission blocking vaccines are difficult to express in the proper conformation
Lack of R&D funding and commercial uncertainty	• Insufficient investment because of an absence of lucrative high-income markets

Unfortunately, because strains in any given locale are genetically and antigenically diverse and variation may occur during the course of an infection, the host must development immunity against several different strains and antigenic variants to be fully protected. In highly endemic areas broad immunity may develop within a few years because of repeated exposure but it is slow to develop in areas of low malaria prevalence (24). The complex life cycle and associated epidemiologic and immunologic peculiarities of malaria combined with challenges inherent to vaccine development underscore why a malaria vaccine with an optimal profile is still a decade or more away.

**Figure 4:**
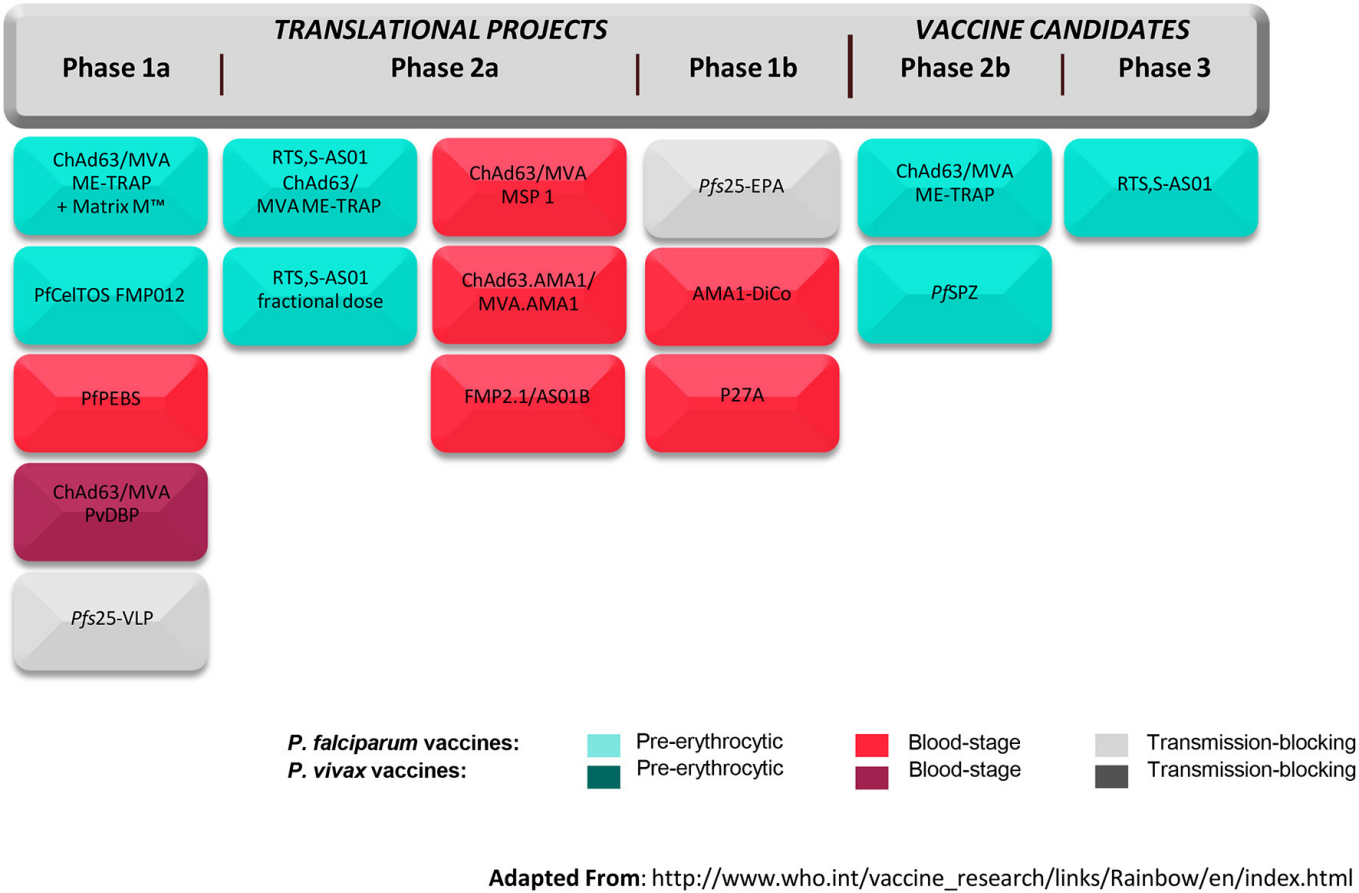
Global malaria vaccine pipeline (July 2017). ChAd63/MVA, Chimpanzee Adenovirus 63 and Modified Vaccinia Virus Ankara; ME-TRAP, fusion protein of a Multi-Epitope string followed by the pre-erythrocytic Thrombospondin Related Adhesion Protein; Matrix M™, proprietary adjuvant; PfCelTOS FMP012, *Plasmodium falciparum* Cell-Traversal Protein for Ookinetes and Sporozoites and Falciparum Malaria Protein 12; PfPEBS, *P. falciparum* Pre-Erythrocytic and Blood Stage; PvDBP, *P. vivax* Duffy-Binding Protein; *Pfs*25-VLP, *P. falciparum* surface protein 25 Virus-Like Particle; RTS,S-AS01, Circumsporozoite protein fused to hepatitis B surface antigen in propriety adjuvant; MSP 1, Malaria Surface Protein 1; AMA1, Apical Membrane Antigen 1; FMP2.1/AS01B, protein based on AMA1 in proprietary adjuvant; *Pfs*25-EPA, *P. falciparum* surface protein 25 conjugated to *Pseudomonas aeruginosa* ExoProtein A; AMA1-DiCo, AMA1 Diversity Covering; P27A, unstructured synthetic peptide from *P. falciparum* trophozoite exported protein 1; *Pfs*SPZ, radiation-attenuated whole organism *P. falciparum* sporozoite. Adapted from the World Health Organization (28).

The global malaria vaccine development strategy to date has been driven by two things—the global health goals for vaccine development and the likelihood of arresting the parasite at a given stage of its life cycle. The Malaria Vaccine Technology Roadmap calls for two objectives to be reached by 2030: (1) Licensure of vaccines against *Plasmodium falciparum* and *Plasmodium vivax* that have at least 75% efficacy against clinical malaria and that are feasible to implement in the real-world setting; and (2) Development of vaccines that will reduce transmission of the parasite, which are suitable for administration in mass campaigns (27). In the context of these objectives, vaccines are being developed that target different stages of the parasite life cycle including the pre-erythrocytic-stage, blood-stage, and sexual-stage. The pre-erythrocytic stage has been the one most popularly targeted; avoiding progression in hepatocytes and erythrocytes seems like a viable immunological and clinical approach. However, other approaches are being pursued in parallel given the limited efficacy of RTS,S/AS01. One advantage that the malaria vaccine development community has over that of other vaccines is the Controlled Human Infection Model (CHIM) in which novel vaccines can be evaluated for efficacy and immunologic correlates of protection in small studies and de-risked before moving into large field trials. In this standardized model, healthy volunteers are given a vaccine and subsequently infected with malaria parasites either through mosquito bites or through injection of sporozoites. The volunteers are monitored for development of malaria parasitemia and any infections curtailed with antimalarial drugs. An early signal of vaccine efficacy may be detected by comparing infection rates in vaccine and placebo recipients. Figure 4 from the World Health Organization summarizes the global malaria vaccine pipeline by phase of development and by stage of the parasite life cycle.

What is the path forward given the ambitious goals but technical uncertainties? First, it is important to build on the knowledge that has been generated from RTS,S/AS01 vaccine studies. It appears that functional antibody against the NANP repeats of CSP are important for protection, and that very high antibody titers persisting for several malaria seasons will be necessary to eliminate disease. Improvements in antibody quantity and quality through the use of novel adjuvants, structure-guided immunogen design, and optimized vaccine regimens will be important for “next-generation” pre-erythrocytic CSP vaccines. Further, given the evidence that antibody may also be important for blood-stage and sexual-stage (transmission blocking) vaccines, taking a similarly rigorous approach in antigen identification and design and using novel adjuvants early may be important lessons learned from pre-erythrocytic vaccines. For liver-stage vaccine candidates, ensuring that the vaccine reaches the hepatocytes that are infected and induces sufficient antigen-specific CD8+ T cell responses will be important, recognizing that novel formulations and routes of administration (e.g., intravenous) may be required. Regardless of the approach, rigor in the development and optimization of assays be they for antigen down-selection or interrogating the immune response are critical. Further, engaging early with experts in protein expression systems and manufacturing process development and scale-up may help the vaccinologist “get ahead” in the development process and be better positioned to move forward robustly with CHIM studies and clinical development when preclinical data are favorable.

## HIV

Since the beginning of the epidemic, 75 million people have been infected with human immunodeficiency virus (HIV) and 32 million people have died (29). Africa is the most severely affected region where nearly one of every 25 individuals is living with HIV. Although pre-exposure prophylaxis (PrEP) is highly effective, it is estimated that the virus is suppressed in just over half of HIV-infected individuals (30). Additional tools such as long-acting drugs, broadly neutralizing antibodies, and vaccines are needed to stop HIV transmission.

The technical challenges of HIV vaccine development have a similar root cause as the challenges for TB and malaria vaccines. First is the virus life cycle. It rapidly establishes chronic infection integrating into the host genome as a DNA provirus, allowing it to escape immune detection. Second, it is tropic for T helper cells which facilitate spread and persistence of the infection. Further, the immunologic mechanism of protection is still being defined. For most viruses, neutralizing antibody is important to prevent entry into the cell. In the case of HIV, each virus is unique and only antibody directed against epitopes conserved across strains will be broadly effective. No vaccine has induced broadly neutralizing antibodies to date (31).

Other challenges not often addressed in the scientific literature are those common to all vaccines in development. For example, expressing trimers of HIV's envelope (Env) glycoprotein (gp) in the proper conformation and manufacturing without proteolytic damage at sufficient yields for supplying clinical trials has been a significant challenge for clinical HIV vaccine research. Further, availability of novel adjuvants to increase the magnitude, breadth, and duration of antibody responses has been limited.

Among its counterpart vaccines discussed in this paper, more progress has been made on HIV vaccine development than in any other area. The scientific learnings from early efficacy trials were sobering but valuable, showing that a subunit Env vaccine, such as the bivalent gp120, was not efficacious and that an adenovirus vector-based vaccine increased the risk of HIV infection ostensibly through activation of CD4+ T cells for which the virus is tropic (31). However, the RV144 study, which showed ~30% efficacy of a vaccine regimen including canary poxvirus prime (ALVAC) followed by an ALVAC + bivalent gp120 boost, gave the first glimpse into immunologic correlates of protection (32). Although not expected, non-neutralizing antibody that bound specific regions of Env correlated with a reduced risk of HIV infection (33).

What is the path forward for HIV vaccines? First, the RV144 efficacy study is being repeated in South Africa with an appropriately matched Clade C vaccine and an additional booster dose. This will provide an opportunity to prospectively confirm the immunologic responses correlated with protection afforded by the vaccine. An efficacy study of another vectored vaccine, adenovirus 26 expressing different mosaic Env/Gag/Pol antigens intended to increase the breadth of immune response followed by a gp140 Env boost is also underway.

An exciting evolution in the field is the identification of a growing number of broadly neutralizing antibodies (bnAbs) that neutralize a large proportion of HIV libraries. Two studies evaluating the efficacy of passively transfused bnAbs against VRC01 to prevent HIV infection are underway. Additional work to further increase breadth, potency, and half-life are ongoing. If the data from the efficacy studies are favorable, bnAbs could both be given to prevent HIV infection and help inform the design of future vaccines with the appropriate antigens needed for protection. To avoid delays, multiple forms of Env are being evaluated for induction of bnAbs and pnnAbs in parallel.

Although the HIV field has a leg up on that of TB and malaria given the insights on immune responses that correlate with disease risk, the fundamental challenges are the same. For transfused bnAbs, will the cost of manufacturing and the need for skilled personnel to administer them preclude widespread use in the regions with the greatest HIV burden? For new HIV vaccines, what is the right antigen(s) needed to induce the desired, broadly neutralizing antibody profile? What are the antigenic conformations, vaccine regimens and/or adjuvants that can ensure that response is long-lived? It is hoped that the results of the aforementioned clinical HIV vaccine and bnAbs trials will shed more light on these questions and surprise us with new knowledge in the ongoing search for an affordable and pragmatic approach for HIV vaccination globally.

## Other Vaccines of Importance for Low Resource Settings

Despite the introduction of pneumococcal conjugate and rotavirus vaccines, lower respiratory tract infections and diarrheal diseases are still in the top ten causes of mortality in low resource settings, with the greatest impact on infants and young children <5 years old (1). In regions where healthcare centers with supportive care are not widely available, vaccines may be the only realistic alternative to address these maladies for decades to come.

### Respiratory Syncytial Virus Vaccine

A leading cause of lower respiratory tract disease behind *Streptococcus pneumoniae* is respiratory syncytial virus (RSV), estimated to cause 33 million cases and 118,000 deaths annually (34). The vast majority of deaths occur in infants in low resources settings; half occur in those 0 to 6 months of age. There is no available treatment for this disease. A monoclonal antibody directed against the fusion protein (F) is available for prevention of severe disease in those at high risk such as preterm infants and infants with congenital heart disease but is unaffordable for low income countries. A formalin-inactivated whole cell RSV vaccine developed in the mid-1960s was associated with enhanced disease in children who subsequently had their first naturally occurring RSV infection, curbing pursuit of an RSV vaccine for decades (35). However, given the significant disease burden–and armed with a better understanding of the immunopathogenesis of enhanced disease–RSV vaccines are being pursue with renewed vigor. The fusion (F) protein, a surface glycoprotein highly conserved across strains that enables the virus to fuse with respiratory epithelial cells is the leading antigenic target being pursued for vaccines (36). This approach is complicated by the fact that the protein exists in different conformations in the pre- and post-fusion state. Although the pre-fusion conformation exposes more neutralizing epitopes, it is less stable and both conformations are being studied.

For low resource settings, RSV vaccine priorities include: (1) Development of vaccines for maternal immunization during pregnancy leading to trans-placental antibody transfer and prevention of severe RSV disease in neonates and young infants; and (2) Development of vaccines to prevent RSV disease in infants and young children (37). The first phase 3 maternal RSV vaccine study, which evaluated a post-fusion aluminum adjuvanted nanoparticle vaccine, failed to meet its primary endpoint but showed 39.4% (95% CI, 5.3 to 61.2) efficacy at 90 days against moderate-to-severe RSV disease globally with higher efficacy among infants in South Africa (38). The vaccine appeared safe with similar rates of adverse events in vaccine and placebo recipients. Additional maternal RSV vaccines in clinical trials include subunit F proteins engineered to maintain the pre-fusion conformation, which may induce more potent antibody at higher titers.

Vaccines being pursued for older infants and young children include attenuated live viruses and vector-based vaccines coding for surface glycoproteins. In clinical trials to date, live virus vaccines have not been associated with enhanced RSV disease; data for vector-based vaccines are still outstanding. Several other vaccine approaches are being utilized for older adults.

RSV vaccine development is not fraught with the same challenges as TB, malaria, and HIV vaccines in that it has a straightforward life cycle, the target antigen is well-defined, and the protective immune response, neutralizing antibody, is well-known. However, RSV vaccine development carries a different set of challenges including those associated with vaccinating pregnant women. Although substantial progress has been made with maternal immunization programs over the last decade and the WHO and many national health authorities recommend maternal immunization based on the clear benefits for mothers and their infants, vaccine coverage rates remain low in some areas. A recent literature review identified several reasons for low vaccine acceptance among pregnant women, with unfounded concerns about maternal and fetal safety being primary among them (39). Concerns about poor vaccine efficacy, lack of disease awareness, and lack of robust recommendations from healthcare providers were also factors. It is important to note that the reasons for under vaccination among pregnant women differ by stakeholder and by geographic region. For example, healthcare workers were more likely to cite inadequate training, inadequate reimbursement, and increased workload as barriers; whereas, pregnant women in low income countries were more likely to report lack of access as a barrier. In summary, the barriers to maternal immunization are complex. In addition to RSV, other maternal vaccines such as those against Group B Streptococcus, influenza, and pertussis may be important to reduce morbidity and mortality in neonates and young infants in low resource settings. While the global health community continues to conduct studies to establish the safety and efficacy of vaccination in pregnant women and their infants, simultaneous research should be conducted to better understand barriers for healthcare workers and the influence of cultural factor's on a woman's decision to be vaccinated.

### Shigella Vaccines

Shigella is the second leading cause of diarrheal disease deaths behind rotavirus in low income countries, estimated to cause over 200,000 deaths annually (40). The majority of deaths occur in children under 5 years of age, the target population for a shigella vaccine. Observations suggesting the feasibility of a shigella vaccine include the acquisition of natural immunity with repeated infections and efficacy demonstrated in challenge studies of non-human primate and controlled human infection models (CHIM). Similar to malaria, the shigella CHIM is a standardized model in which healthy volunteers are given an investigational vaccine and a standard dose of shigella inoculum orally. Subjects are followed for symptoms that meet a consensus clinical endpoint definition and administered antibiotics according to certain criteria. Assuming favorable results, this model may be helpful to inform and de-risk larger phase 2 and phase 3 clinical efficacy studies. Immunity appears to be strain specific.

Several shigella vaccines are in various stages of development. The leading candidates contain O-specific polysaccharide antigens, sparked by the successful results of a large field trial of a conjugate vaccine composed of *Shigella sonnei* O-specific polysaccharide conjugated to *Pseudomonas aeruginosa* recombinant exoprotein A (*S. sonnei*-r EPA). The vaccine was ~74% efficacious in adults with lower efficacy in young children and no efficacy in those between 1 to 2 years of age (41). The magnitude of binding antibody titers to the O antigen appeared to correlate with protection across the age ranges studied. Currently, shigella conjugate vaccines including chemical, synthetic, and bioconjugates along with live attenuated, inactivated whole cell, conserved subunit proteins, and vesicle-based vaccines are in development.

The challenges of shigella vaccine development are those typical of any vaccine. The first challenge is strain variation and the need for a multi-valent vaccine. Although only four species, there are ~50 serotypes and subserotypes of shigella that cause human disease. Experts agree that a quadrivalent vaccine targeting *Shigella sonnei* and *Shigella flexneri* 2a, 3a, and 6 would cover the majority (~88%) of disease in young children (40, 42). However, this approach will significantly increase the complexity of manufacturing and clinical development.

A second challenge is the lack of clarity on the mechanism(s) by which immunity is generated. Although data from the study of the *S. sonnei*-r EPA vaccine are encouraging, validation of the correlation between binding O-antigen antibody titers and protection have yet to be confirmed, particularly for young children. Reference standards to assist with comparing results across studies and functional assays to evaluate bactericidal activity are under development (40). Further, the low efficacy of the *S. sonnei*-r EPA vaccine in children suggests either an improved conjugate and/or an adjuvant may be needed to induce sufficient protective antibody titers in the target population. It is also possible that a different immunologic mechanism may be responsible for immunity in children in low resource settings, which is yet to be explored.

Finally, requirements for clinical trials and regulatory approval are being evaluated (40). Pivotal registration studies evaluating the prevention of clinically confirmed cases of shigellosis in children under five will be large and operationally complex to implement. The possibility of utilizing data from CHIM studies in combination with other supportive data from adult studies to streamline development and support early registration is under discussion.

## Conclusions

The development of vaccines against diseases that are prevalent almost solely in low resource settings is incredibly challenging, combining the traditional complexities of biologics manufacturing and immunologic characterization with the technical complexities of pathogens with elaborate life cycles and unclear immunology. The vaccines must be studied in populations who live in areas without sufficient clinical trial and laboratory site infrastructure. Further, the global funding envelop for each of these vaccines is limited. This is not an effort for the faint of heart. However, technological advances in high throughput antigen and antibody screening, immuno-profiling, and advanced manufacturing techniques put us in a better position today than ever before to achieve success. In fact, when one looks at the global health vaccine pipeline and the vaccine candidates currently in clinical trials, it is quite feasible that vaccines with sufficient efficacy against TB, malaria, HIV, RSV, and *Shigella* to support widespread use will be available within the next decade. Further, long-acting therapeutics and monoclonal antibodies against malaria, HIV, and RSV that are highly potent, easy to administer, and affordable for low income countries will also likely exist. Our future challenge may be developing a people-centered care approach that sufficiently addresses the needs of individuals and their communities. Simplified manufacturing approaches with modular bioprocess and fill/finish units may make regional manufacturing an affordable model and reduce the need for large capital expenditures. This progress does not mean that basic research on life cycles, antigen selection, and immunopathogenesis should be slowed; in fact, the push should continue to simultaneously develop product candidates that have potential for higher efficacy similar to that observed with other routine childhood vaccines. Nonetheless, it is motivating to reflect on the fact that the tools exist today to move diseases that are currently an everyday fear of individuals living in low resources areas to their rightful place in history–eliminated.

## Author Contributions

PH wrote, revised, reviewed, and approved the final manuscript.

## Conflict of Interest

The author declares that the research was conducted in the absence of any commercial or financial relationships that could be construed as a potential conflict of interest.
